# Translation initiation from sequence variants of the bacteriophage T7 g10RBS in *Escherichia coli* and *Agrobacterium fabrum*

**DOI:** 10.1007/s11033-021-06891-z

**Published:** 2021-11-07

**Authors:** Alex B. Benedict, Joshua D. Chamberlain, Diana G. Calvopina, Joel S. Griffitts

**Affiliations:** Department of Microbiology and Molecular Biology, Brigham Young University, Provo, UT 84602 USA

**Keywords:** Recombinant protein expression, Ribosome binding site, Translation

## Abstract

**Background:**

The bacteriophage T7 gene *10* ribosome binding site (g10RBS) has long been used for robust expression of recombinant proteins in *Escherichia coli*. This RBS consists of a Shine–Dalgarno (SD) sequence augmented by an upstream translational “enhancer” (Enh) element, supporting protein production at many times the level seen with simple synthetic SD-containing sequences. The objective of this study was to dissect the g10RBS to identify simpler derivatives that exhibit much of the original translation efficiency.

**Methods and results:**

Twenty derivatives of g10RBS were tested using multiple promoter/reporter gene contexts. We have identified one derivative (which we call “CON_G”) that maintains 100% activity in *E. coli* and is 33% shorter. Further minimization of CON_G results in variants that lose only modest amounts of activity. Certain nucleotide substitutions in the spacer region between the SD sequence and initiation codon show strong decreases in translation. When testing these 20 derivatives in the alphaproteobacterium *Agrobacterium fabrum*, most supported strong reporter protein expression that was not dependent on the Enh.

**Conclusions:**

The g10RBS derivatives tested in this study display a range of observed activity, including a minimized version (CON_G) that retains 100% activity in *E. coli* while being 33% shorter. This high activity is evident in two different promoter/reporter sequence contexts. The array of RBS sequences presented here may be useful to researchers in need of fine-tuned expression of recombinant proteins of interest.

**Supplementary Information:**

The online version contains supplementary material available at 10.1007/s11033-021-06891-z.

## Introduction

Recombinant protein expression in *Escherichia coli* is often optimized by engineering two control points: transcription (promoter optimization) and translation (ribosome binding site, or RBS, optimization) [1, 2]. The RBS from the bacteriophage T7 gene *10* (g10RBS) has been used extensively in commercially available plasmids to stimulate recombinant protein expression in *E. coli*. It was previously shown that when g10RBS is placed upstream of recombinant genes, protein expression is increased 40-fold or more than when a synthetic consensus RBS sequence is used [3]. Within this 45-nucleotide (nt) sequence, an A/U-rich 9-nt enhancer element (Enh) upstream of the Shine–Dalgarno (SD) region is found to stimulate translation, even when its position relative to the SD region is changed [4]. Several studies have examined the effects of this Enh element on translation rates [3–10]. Homology between the Enh and nucleotides 458–466 in the 16S rRNA led to an initial proposal that base-pairing underlies the increased translation rates [4, 8]. Modifications to nucleotides 458–466 of the 16S rRNA, however, did not support that hypothesis [6]. Subsequent proposals suggest, instead, that Enh or similar elements interact with ribosomal protein S1 [11–13]. Beyond *E. coli*, the g10RBS has been shown to support high expression of recombinant proteins in other gammaproteobacteria such as *Pseudomonas*, *Erwinia*, and *Serratia* [14]. The objective of the present study is to more thoroughly dissect this phage-derived RBS to identify shorter derivatives that retain translational activity in *E. coli*, and to test these derivatives in the more distantly related alphaproteobacterial species, *Agrobacterium fabrum*.

## Results and discussion

**Fig. 1:**
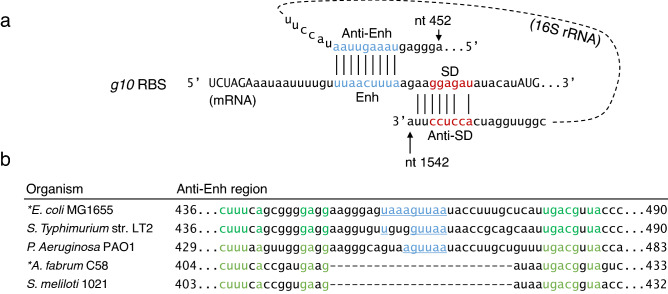
Homology between g10RBS and two positions within the 16S rRNA. **a** Blue and red coloring indicates the Enh/Anti-Enh and SD/Anti-SD sequences, respectively. **b** Alignment of the anti-Enh region in several species. Green coloring indicates residues conserved in all species and blue, underlined, text indicates homology with the Enh. (Color figure online)

The full-length g10RBS used in commercial vectors to enhance translation is referred to in this study as “FL_A,” which includes (from the 5’ end) an XbaI site, an 11-nt A/U-rich sequence, the 9-nt Enh element, a 4-nt potential “standby” site, the SD sequence (GGAGAT), and the SD-initiation codon spacer. The g10RBS and its potential to hybridize with the *E. coli* 16S rRNA are depicted in Fig. 1a. The anti-Enh region within the 16S sequence is not found in the alphaproteobacterial rRNAs from *A. fabrum* and *Sinorhizobium meliloti* (Fig. 1b).

**Fig. 2:**
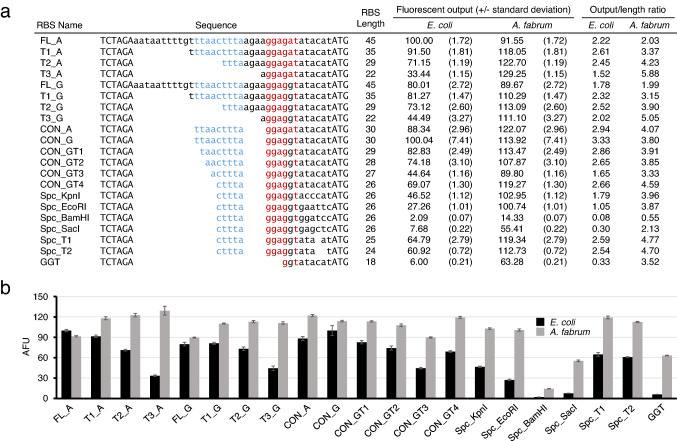
Effects of RBS modifications on fluorescent output. **a** Sequence and length of each RBS is displayed along with its relative strength. Output/length ratio was calculated by dividing the relative strength value by the length of the RBS sequence. **b** Relative fluorescence in each strain was determined by using the average of nine cultures (three technical replicates from each of three biological replicates). The wildtype g10RBS (FL_A) measurement from *E. coli* was calibrated to “100” with all other fluorescence values normalized to that standard. Error bars indicate the standard deviation

A total of 21 RBS sequences, including FL_A, were ligated individually into a test plasmid that replicates in both *E. coli* and *A. fabrum*, and encodes the mScarlet-I fluorescent protein [15], driven by a synthetic *lac*T5 promoter. Because the plasmid does not encode the LacI repressor, the *lac*T5 promoter behaves constitutively in these strains. Fluorescent output for each RBS was measured in *E. coli* and *A. fabrum* (Fig. 2) and are presented as normalized values with the FL_A measurement in *E. coli* calibrated to “100”. All other fluorescence measurements presented are normalized to that standard.

The SD sequence within FL_A (GGAGAT) does not match perfectly with the anti-SD at the 3’ end of 16S in *E. coli* or *A. fabrum*, so the A nucleotide was changed to G to make a perfect match (GGAGGT). We refer to this derivative as “FL_G.” For FL_G and FL_A, a series of three 5’ truncations were made. Next, the 11-nt A/U-rich sequence upstream of Enh and the 4-nt potential standby site were simultaneously removed from FL_A and FL_G to form a condensed RBS. These are called “CON_A” and “CON_G”, respectively. Then, four single-base truncations were made from the 5’ end of Enh in the FL_G derivative. Finally, in the derivative with 4 bases truncated from the 5’ end of Enh (termed “CON_GT4”), we made several modifications to the spacer between the SD and the initiation codon by either removing bases or replacing them to incorporate various restriction sites (see Fig. 2).

In *E. coli*, the A-to-G base substitution in the SD sequence (FL_A vs FL_G) led to a 20% drop in expression. Each of the three truncations within the FL_A sequence (“T1_A”, “T2_A”, and “T3_A”) led to 9%, 29%, and 67% reductions in expression, respectively. In contrast, the same three truncations within the FL_G sequence (“T1_G”, “T2_G”, and “T3_G”) led to a 2% increase, and 9% and 44% decreases, respectively. When comparing CON_A and CON_G to FL_A, we observed, surprisingly, that the CON_G derivative yielded expression levels nearly identical to the parental FL_A sequence despite being 33% (15 nt) shorter, while expression from the CON_A derivative was 12% lower than FL_A. This observation is consistent with data from two high-throughput studies on the 5’ untranslated regions of mRNAs in *E. coli*, where it was determined that there is a preference for a G nucleotide eight bases upstream of the initiation codon [16, 17]. For this reason, subsequent RBS modifications were made to the highly efficient CON_G sequence. Four single-base deletions from the 5’ end of the enhancer element in CON_G (“CON_GT1”, “CON_GT2”, “CON_GT3”, and “CON_GT4”) led to 17%, 26%, 55%, and 31% reductions in expression, respectively. Modifying the spacer between the SD and the initiation codon to incorporate restriction sites (“Spc_KpnI”, “Spc_EcoRI”, “Spc_BamHI”, and “Spc_SacI”) led to 33%, 61%, 97%, and 89% reductions in expression, respectively. Removing 1 and 2 bases (“Spc_T1” and “Spc_T2”) from the spacer led to 6% and 12% reductions in expression, respectively (see Fig. 2).

In *A. fabrum*, FL_A and FL_G gave nearly identical expression levels and truncations of these yielded even higher levels of translation. In fact, the 22-nt T3_A sequence supported the highest level of translation of all 21 sequences tested. The CON_A and CON_G RBS sequences yielded 33% and 24% (respectively) more fluorescence than FL_A, with CON_A exhibiting 94% of the activity as T3_A. The CON_GT series of Enh truncations had similar effects in *A. fabrum* as in *E*. *coli*, with the CON_GT4 variant unexpectedly giving higher expression than CON_GT3. Levels of expression in *A. fabrum* remained high in most cases when modifying the spacer between the SD and initiation codon, two exceptions being the Spc_BamHI and Spc_SacI variants, which led to 88% and 54% decreases in expression, respectively. These are also the variants that gave the lowest expression values in *E*. *coli*. Interestingly, cultures of *A. fabrum* harboring a few of the most active RBS sequences (T1_A, T2_A, T3_A, and CON_A) initially had high levels of fluorescence and somewhat decreased cell density. In subsequent passages of these cultures, normal cell density was restored, but fluorescence decreased—suggesting a fitness cost associated with such high-level expression of the reporter gene. Consistent with this, colonies from these high-expressing strains appeared slightly smaller compared to the other strains (see Supplementary Fig. S1a–c). Suppression of fitness defects was possibly due to mutations in the promoter, RBS, reporter gene, or plasmid origin of replication, though the precise cause was not investigated.

**Fig. 3:**
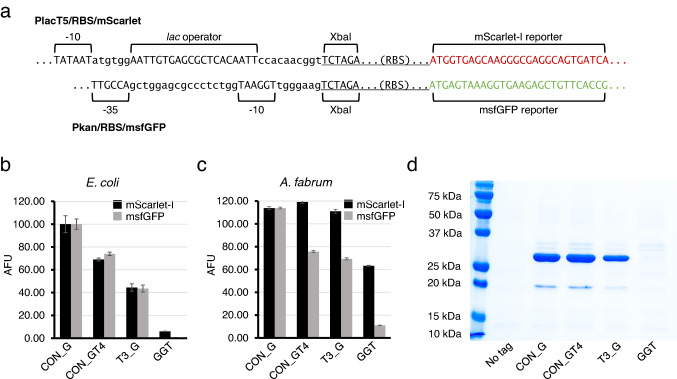
Effects of selected RBS sequences on fluorescent output in a new promoter/reporter gene context. **a** The two RBS sequence contexts are displayed. **b** and **c** Relative mScarlet and GFP fluorescence in each strain was determined by using the average of nine cultures (three technical replicates from each of three biological replicates). Error bars indicate the standard deviation. **d** His_6_ tagged GFP was purified from *E. coli* cells harboring the selected RBS sequences

To determine if the effect of RBS variants on reporter gene expression is in part due to flanking sequences, we selected four RBS variants to test in a new promoter/reporter context. The RBS sequences were selected to represent a wide range of translation enhancement. Whereas the original system employs the *lac*T5 promoter and mScarlet-I reporter, the alternative system uses the constitutive Pkan promoter (from the Tn5 transposon) and a monomeric superfolder GFP (msfGFP) reporter. The dissimilarity of flanking sequences in these two systems is shown in Fig. 3a. In *E. coli*, no differences in relative RBS activity could be seen between these sequence contexts (Fig. 3b). In *A. fabrum*, the relative values differ somewhat. For example, CON_GT4 gives slightly higher translation than CON_G in the mScarlet-I context (a ~ 5% increase), but substantially lower activity in the msfGFP context (a 33% decrease; see Fig. 3c).

To test the influence of the four RBS variants on protein expression and purification from *E. coli*, the msfGFP gene was modified to encode a *C-*terminal His_6_ tag in each expression plasmid. Expression and purification according to conventional methods (see Materials and Methods) was carried out, and gel analysis of purified products is shown in Fig. 3d. Consistent with the fluorescence-based analysis, CON_G and CON_GT4 gave the highest yield of recombinant protein, with T3_G yielding a lower amount, and GGT yielding undetectable levels of recombinant protein.

Among the most notable RBS variants tested in this study, the SD-initiator spacer variants were rather surprising, though consistent with recent work indicating that adenosine nucleotides in this region can enhance translation, while cytidine nucleotides are unfavorable [18]. The most detrimental spacer substitutions in our analysis were Spc_SacI (ATACAT to GAGCTC) and Spc_BamHI (ATACAT to GGATCC). Both of these changes reduce the number of A nucleotides and increase the number of C nucleotides. We acknowledge, however, that the effect of spacer sequence on translation efficiency is idiosyncratic, as recent high-throughput studies identified efficient RBS sequences that deviate from this rule [19, 20].

In both *E. coli* and *A. fabrum*, we expected the CON_GT1/2/3/4 truncation series (which removes 1 to 4 nucleotides from Enh) to exhibit a downward trend in expression level compared to CON_G. While this was partly true, CON_GT4 showed a rebounded level of expression compared to CON_GT3. This observation bolsters the model in which the effect of Enh does not necessarily depend on its propensity to hybridize to the complementary sequence on 16S [6]. High levels of expression in *A. fabrum* for T3_A and T3_G indicate the dispensability of Enh in this organism.

The g10RBS derivatives tested in this study display a range of observed activity, including a minimized version (CON_G) that retains 100% activity in *E. coli* while being 33% shorter. This high activity is evident in two different promoter/reporter sequence contexts. *A. fabrum* appears to be less influenced by the varying RBS sequences tested here which may be related to the parental sequence not being native to this species. The set of RBS sequences presented here may be useful to researchers in need of fine-tuned expression of recombinant proteins of interest in diverse species.

## Materials and methods

### Bacterial genetic manipulations and growth conditions

*Escherichia coli* DH5α (Sharon Long collection) and *A. fabrum* B527 (a streptomycin-resistant derivative of C58, Griffitts lab collection) strains were grown in lysogeny broth (LB) at 37 °C or 30 °C, respectively. Fluorescent protein expression plasmids were constructed in vitro, transformed into DH5α, sequence-verified, and transferred to *A. fabrum* by conjugation using helper strain B001 [21]. Plasmid selection was carried out in lysogeny broth (LB) containing 30 μg/ml kanamycin (GoldBio K-120–5) (*E. coli*) or 100 μg/ml neomycin (Sigma N1876-25G) (*A. fabrum*). Full sequences for the FL_A-containing starting plasmids (pJG1082 for mScarlet-I expression and pAB215 for msfGFP expression) are given as supplementary information, with the g10RBS and initiation codon capitalized. Variants of these plasmids, and primers used for their construction, are documented in Supplementary Tables S1 and S2.

### Measurement of fluorescent output

*Escherichia coli* and *A. fabrum* strains harboring plasmids with modified RBS sequences were used to start liquid LB cultures, which were initially grown to saturation. Saturated cultures were passaged in triplicate to fresh LB and grown for 8 h (*E. coli*) or 19 h (*A. fabrum*) before taking OD_600_ measurements and adjusting all cultures to an OD of 0.5. After adjustment, each culture was then distributed in triplicate to a flat-bottom, 96-well microplate (CellStar, F-bottom), and fluorescent measurements were taken on a VICTOR Nivo Multimode Plate Reader. For mScarlet-I measurements, the excitation and emission filters were set to 540/10 nm and 595/10 nm, respectively; for msfGFP measurements, the excitation and emission filters were set to 480/30 nm and 530/30 nm, respectively. Negative control strains lacking fluorescent reporters were used to subtract background fluorescence. The wildtype FL_A measurement in *E. coli* was calibrated to “100” and all other fluorescence measurements were normalized to that standard.

### Protein purification

To prepare samples for msfGFP-His_6_ purification, 100-ml cultures were inoculated with 2 ml of overnight culture and allowed to grow at 30 °C for 7.5 h. Centrifuged cell pellets were frozen at − 80 °C overnight. Pellets were then thawed on ice and re-suspended in 1.3 ml of lysis buffer (50 mM HEPES pH 7.8 (FisherScientific BP310-500), 300 mM NaCl (Mallinckrodt Chemicals 7581-06), 0.2% Triton X-100 (Sigma T8787-100 ml), 0.5 mg/ml lysozyme (Sigma L6876-5G), 60 mM imidazole (Sigma 12399-100G), 1 mM EDTA (Sigma E4884-500G)). Lysis took place for 1 h at 4 °C. Cell lysates were then sonicated to ensure complete lysis and fragmentation of DNA. Sonicated samples were cleared by centrifugation, and supernatant transferred to a new microcentrifuge tube. This was incubated end-over-end with 50 μl of NTA-nickel agarose beads (Qiagen 1018244) at 4 °C for 30 min. Beads were washed four times with 1 ml wash buffer (50 mM HEPES pH 7.8, 300 mM NaCl, and 60 mM imidazole), and protein was eluted by incubating the beads in 50 μl of SDS-containing sample buffer at 100 °C. Eluted protein samples were resolved by SDS-PAGE (12% acrylamide, Apex Bioresearch Products 18–197) and stained using Coomassie blue prior to imaging.

## Supplementary Information

Below is the link to the electronic supplementary material.

Supplementary file1 Effects of high reporter-protein expression on *A*. *fabrum*. Cultures were grown in triplicate to saturation then, at each passage, cell density was normalized before taking fluorescence measurements. OD-adjusted cultures were then transferred to fresh media and the process was repeated until Passage 4. Error bars indicate the standard deviation. **a** mScarlet-I fluorescence measurements for each culture were taken, in triplicate, at each passage (nine total measurements per strain/per passage). **b** OD_600_ values at each passage were measured in triplicate (nine total measurements per strain/per passage). **c** Representative pictures of *A*. *fabrum* colonies (from several separate experiments) after selecting for mScarlet-I reporter expression plasmids harboring the specified RBS derivative. Scale bars represent 0.5 cm (PPTX 1925 kb)

Supplementary file2 (DOCX 23 kb)

## Data Availability

All data used for figures can be made available upon request.
