# Supplementary material for: Translation initiation from sequence variants of the bacteriophage T7 g10RBS in Escherichia coli and Agrobacterium fabrum
Source: Mol Biol Rep. 2021 Nov 7;49(1):833–8. doi: 10.1007/s11033-021-06891-z (PMC8748333; doi:10.1007/s11033-021-06891-z)
Supplement: Supplementary file 1 — Supplementary file1 Effects of high reporter-protein expression on A. fabrum. Cultures were grown in triplicate to saturation then, at each passage, cell density was normalized before taking fluorescence measurements. OD-adjusted cultures were then transferred to fresh media and the process was repeated until Passage 4. Error bars indicate the standard deviation. a mScarlet-I fluorescence measurements for each culture were taken, in triplicate, at each passage (nine total measurements per strain/per passage). b OD600 values at each passage were measured in triplicate (nine total measurements per strain/per passage). c Representative pictures of A. fabrum colonies (from several separate experiments) after selecting for mScarlet-I reporter expression plasmids harboring the specified RBS derivative. Scale bars represent 0.5 cm (PPTX 1925 kb) [file 11033_2021_6891_MOESM1_ESM.pptx]

## Slide 1
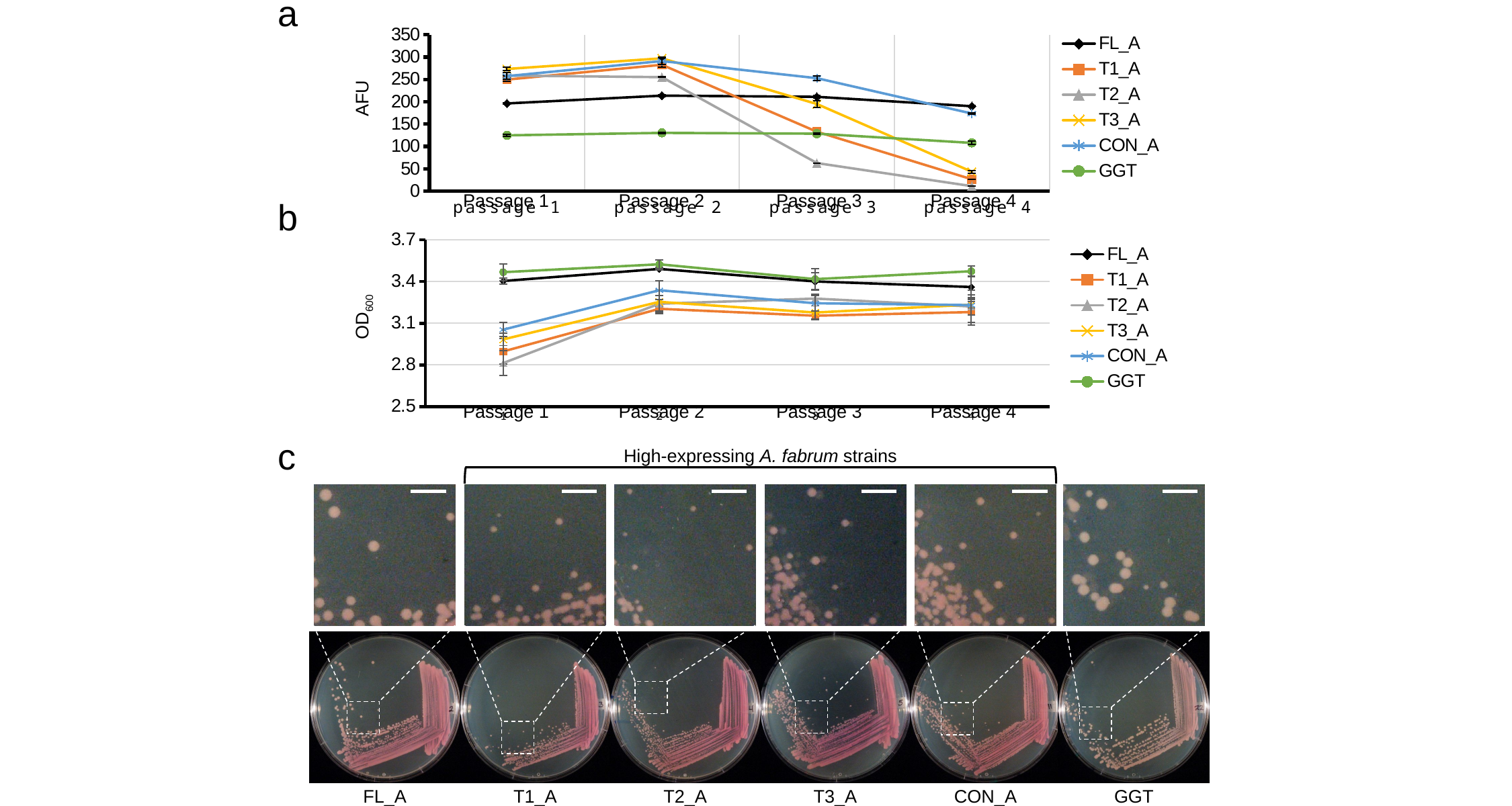

a
### Chart
| Category | | | | | | |
|---|---|---|---|---|---|---|
| passage 1 | 196.3436389 | 249.64132478888885 | 258.6069021222222 | 273.47198956666665 | 257.4614620111111 | 124.90541656666667 |
| passage 2 | 213.84266388999998 | 282.85048766777777 | 255.2113022233333 | 297.31950811222225 | 291.2249835566666 | 130.4233817788889 |
| passage 3 | 211.19164377333334 | 133.00854888444442 | 62.887639217777775 | 195.11126988444443 | 252.86253443999996 | 128.5987335511111 |
| passage 4 | 190.14865088666664 | 26.59065666444444 | 11.265458664444443 | 43.427172775555555 | 173.77826810888888 | 107.97538888666665 |AFU
Passage 1
Passage 2
Passage 3
Passage 4
b
### Chart
| Category | | | | | | |
|---|---|---|---|---|---|---|OD600
Passage 1
Passage 2
Passage 3
Passage 4
c
High-expressing A. fabrum strains
FL_A
T1_A
T2_A
T3_A
CON_A
GGT
