# Supplementary material for: Translation initiation from sequence variants of the bacteriophage T7 g10RBS in Escherichia coli and Agrobacterium fabrum
Source: Mol Biol Rep. 2021 Nov 7;49(1):833–8. doi: 10.1007/s11033-021-06891-z (PMC8748333; doi:10.1007/s11033-021-06891-z)
Supplement: Supplementary file 2 — Supplementary file2 (DOCX 23 kb) [file 11033_2021_6891_MOESM2_ESM.docx]

**Supplementary Information**

**Molecular Biology Reports**

**Translation initiation from sequence variants of the bacteriophage T7 g10RBS in *Escherichia coli* and *Agrobacterium fabrum***

Alex B. Benedict^1^, Joshua D. Chamberlain^1^, Diana G. Calvopina^1^, *Joel S. Griffitts^1^

^1^Department of Microbiology and Molecular Biology, Brigham Young University, Provo, Utah, 84602, USA

*Corresponding author:

Email: joelg@byu.edu

Phone: 801-422-7997

pJG1082 plasmid sequence (5908 bp – g10RBS and initiation codon capitalized):

gagctcaccgaattcccactcgagccaggatccccggtgagatctcgatcccgcgaaataattgtgagcggataacaattacgagcttcatgcacagtgaaatcatgaaaaatttatttgctttgtgagcggataacaattataatatgtggaattgtgagcgctcacaattccacaacggtTCTAGAAATAATTTTGTTTAACTTTAAGAAGGAGATATACATATGgtgagcaagggcgaggcagtgatcaaggagttcatgcggttcaaggtgcacatggagggctccatgaacggccacgagttcgagatcgagggcgagggcgagggccgcccctacgagggcacccagaccgccaagctgaaggtgaccaagggtggccccctgcccttctcctgggacatcctgtcccctcagttcatgtacggctccagggccttcatcaagcaccccgccgacatccccgactactataagcagtccttccccgagggcttcaagtgggagcgcgtgatgaacttcgaggacggcggcgccgtgaccgtgacccaggacacctccctggaggacggcaccctgatctacaaggtgaagctccgcggcaccaacttccctcctgacggccccgtaatgcagaagaagacaatgggctgggaagcgtccaccgagcggttgtaccccgaggacggcgtgctgaagggcgacattaagatggccctgcgcctgaaggacggcggccgctacctggcggacttcaagaccacctacaaggccaagaagcccgtgcagatgcccggcgcctacaacgtcgaccgcaagttggacatcacctcccacaacgaggactacaccgtggtggaacagtacgaacgctccgagggccgccactccaccggcggcatggacgagctgtacaagtaagcttccacagcaagcgaaccggaattgccagctggggcgccctctggtaaggttgggaagccctgcaaagtaaactggatggctttcttgccgccaaggatctgatggcgcaggggatcaagatctgatcaagagacaggatgaggatcgtttcgcatgattgaacaagatggattgcacgcaggttctccggccgcttgggtggagaggctattcggctatgactgggcacaacagacaatcggctgctctgatgccgccgtgttccggctgtcagcgcaggggcgcccggttctttttgtcaagaccgacctgtccggtgccctgaatgaactgcaggacgaggcagcgcggctatcgtggctggccacgacgggcgttccttgcgcagctgtgctcgacgttgtcactgaagcgggaagggactggctgctattgggcgaagtgccggggcaggatctcctgtcatctcaccttgctcctgccgagaaagtatccatcatggctgatgcaatgcggcggctgcatacgcttgatccggctacctgcccattcgaccaccaagcgaaacatcgcatcgagcgagcacgtactcggatggaagccggtcttgtcgatcaggatgatctggacgaagagcatcaggggctcgcgccagccgaactgttcgccaggctcaaggcgcgcatgcccgacggcgaggatctcgtcgtgacccatggcgatgcctgcttgccgaatatcatggtggaaaatggccgcttttctggattcatcgactgtggccggctgggtgtggcggaccgctatcaggacatagcgttggctacccgtgatattgctgaagagcttggcggcgaatgggctgaccgcttcctcgtgctttacggtatcgccgctcccgattcgcagcgcatcgccttctatcgccttcttgacgagttcttctgacccggtacctcagcgctagcggagtgtatactggcttactatgttggcactgatgagggtgtcagtgaagtgcttcatgtggcaggagaaaaaaggctgcaccggtgcgtcagcagaatatgtgatacaggatatattccgcttcctcgctcactgactcgctacgctcggtcgttcgactgcggcgagcggaaatggcttacgaacggggcggagatttcctggaagatgccaggaagatacttaacagggaagtgagagggccgcggcaaagccgtttttccataggctccgcccccctgacaagcatcacgaaatctgacgctcaaatcagtggtggcgaaacccgacaggactataaagataccaggcgtttccccctggcggctccctcgtgcgctctcctgttcctgcctttcggtttaccggtgtcattccgctgttatggccgcgtttgtctcattccacgcctgacactcagttccgggtaggcagttcgctccaagctggactgtatgcacgaaccccccgttcagtccgaccgctgcgccttatccggtaactatcgtcttgagtccaacccggaaagacatgcaaaagcaccactggcagcagccactggtaattgatttagaggagttagtcttgaagtcatgcgccggttaaggctaaactgaaaggacaagttttggtgactgcgctcctccaagccagttacctcggttcaaagagttggtagctcagagaaccttcgaaaaaccgccctgcaaggcggttttttcgttttcagagcaagagattacgcgcagaccaaaacgatctcaagaagatcatcttattaaggggtctgacgctcagtggaacgaaaactcacgttaagggattttggtcatgagattatcaaaaaggatcttcacctagatccttttaaattaaaaatgaagttttaaatcaatctaaagtatatatgagtaaacttggtctgacagttaccaatgcttaatcagactagagcttccatccgcttgccctcatctgttacgccggcggtagccggccagcctcgcagagcaggattcccgttgagcaccgccaggtgcgaataagggacagtgaagaaggaacacccgctcgcgggtgggcctacttcacctatcctgcccggctgacgccgttggatacaccaaggaaagtctacacgaaccctttggcaaaatcctgtatatcgtgcgaattgatccaccgtgcggctgcatgaaatcctggccggtttgtctgatgccaagctggcggcctggccggccagcttggccgctgaagaaaccgagcgccgccgtctaaaaaggtgatgtgtatttgagtaaaacagcttgcgtcatgcggtcgctgcgtatatgatgcgatgagtaaataaacaaatacgcaaggggaacgcatgaaggttatcgctgtacttaaccagaaaggcgggtcaggcaagacgaccatcgcaacccatctagcccgcgccctgcaactcgccggggccgatgttctgttagtcgattccgatccccagggcagtgcccgcgattgggcggccgtgcgggaagatcaaccgctaaccgttgtcggcatcgaccgcccgacgattgaccgcgacgtgaaggccatcggccggcgcgacttcgtagtgatcgacggagcgccccaggcggcggacttggctgtgtccgcgatcaaggcagccgacttcgtgctgattccggtgcagccaagcccttacgacatatgggccaccgccgacctggtggagctggttaagcagcgcattgaggtcacggatggaaggctacaagcggcctttgtcgtgtcgcgggcgatcaaaggcacgcgcatcggcggtgaggttgccgaggcgctggccgggtacgagctgcccattcttgagtcccgtatcacgcagcgcgtgagctacccaggcactgccgccgccggcacaaccgttcttgaatcagaacccgagggcgacgctgcccgcgaggtccaggcgctggccgctgaaattaaatcaaaactcatttgagttaatgaggtaaagagaaaatgagcaaaagcacaaacacgctaagtgccggccgtccgagcgcacgcagcagcaaggctgcaacgttggccagcctggcagacacgccagccatgaagcgggtcaactttcagttgccggcggaggatcacaccaagctgaagatgtacgcggtacgccaaggcaagaccattaccgagctgctatctgaatacatcgcgcagctaccagagtaaatgagcaaatgaataaatgagtagatgaattttagcggctaaaggaggcggcatggaaaatcaagaacaaccaggcaccgacgccgtggaatgccccatgtgtggaggaacgggcggttggccaggcgtaagcggctgggttgtctgccggccctgcaatggcactggaacccccaagcccgaggaatcggcgtgacggtcgcaaaccatccggcccggtacaaatcggcgcggcgctgggtgatgacctggtggagaagttgaaggccgcgcaggccgcccagcggcaacgcatcgaggcagaagcacgccccggtgaatcgtggcaagcggccgctgatcgaatccgcaaagaatcccggcaaccgccggcagccggtgcgccgtcgattaggaagccgcccaagggcgacgagcaaccagattttttcgttccgatgctctatgacgtgggcacccgcgatagtcgcagcatcatggacgtggccgttttccgtctgtcgaagcgtgaccgacgagctggcgaggtgatccgctacgagcttccagacgggcacgtagaggtttccgcagggccggccggcatggccagtgtgtgggattacgacctggtactgatggcggtttcccatctaaccgaatccatgaaccgataccgggaagggaagggagacaagcccggccgcgtgttccgtccacacgttgcggacgtactcaagttctgccggcgagccgatggcggaaagcagaaagacgacctggtagaaacctgcattcggttaaacaccacgcacgttgccatgcagcgtacgaagaaggccaagaacggccgcctggtgacggtatccgagggtgaagccttgattagccgctacaagatcgtaaagagcgaaaccgggcggccggagtacatcgagatcgagctagctgattggatgtaccgcgagatcacagaaggcaagaacccggacgtgctgacggttcaccccgattactttttgatcgatcccggcatcggccgttttctctaccgcctggcacgccgcgccgcaggcaaggcagaagccagatggttgttcaagacgatctacgaacgcagtggcagcgccggagagttcaagaagttctgtttcaccgtgcgcaagctgatcgggtcaaatgacctgccggagtacgatttgaaggaggaggcggggcaggctggcccgatcctagtcatgcgctaccgcaacctgatcgagggcgaagcatccgccggttcctaatgtacggagcagatgctagggcaaattgccctagcaggggaaaaaggtcgaaaaggtctctttcctgtggatagcacgtacattgggaacccaaagccgtacattgggaaccggaacccgtacattgggaacccaaagccgtacattgggaaccggtcacacatgtaagtgactgatataaaagagaaaaaaggcgatttttccgcctaaaactctttaaaacttattaaaactcttaaaacccgcctggcctgtgcataactgtctggccagcgcacagccgaagagctgcaaaaagcgcctacccttcggtcgctgcgctccctacgccccgccgcttcgcgtcggcctatcgcggccgctggccgctcaaaaatggctggcctacggccaggcaatctaccagggcgcggacaagccgcgccgtcgccactcgaccgccggcgcccacatcaaggcaccctgcct

pAB215 plasmid sequence (5937 bp – g10RBS and initiation codon capitalized):

gagctcaccgaattcccactcgagccaggatccccggtgagatctcgatcccgcgaaataattgtgagcggataacaattacgagcttcatgcacagtgaaatcatgaaaaatttatttgctttgtgagcggataacaattataatatgtggaattgtgagcgctcacaattccacaacggtTCTAGAAATAATTTTGTTTAACTTTAAGAAGGAGATATACATATGagtaaaggtgaagagctgttcaccggggtggtgcccatcctggtcgagctggacggcgacgtaaacggccacaagttcagcgtgcgcggcgagggcgagggcgatgccaccaacggcaagctgaccctgaagttcatctgcaccaccggcaagctgcccgtgccctggcccaccctcgtgaccaccctgacctacggcgtgcagtgcttcagccgctaccccgaccacatgaagcagcacgacttcttcaagtccgccatgcccgaaggctacgtccaggagcgcaccatctccttcaaggacgacggcacctacaagacccgcgccgaggtgaagttcgagggcgacaccctggtgaaccgcatcgagctgaagggcatcgacttcaaggaggacggcaacatcctggggcacaagctggagtacaacttcaacagccacaacgtctatatcacggccgacaagcagaagaacggcatcaaggcgaacttcaagatccgccacaacgtcgaggacggcagcgtgcagctcgccgaccactaccagcagaacacccccatcggcgacggccccgtgctgctgcccgacaaccactacctgagcacccagtccaagctgagcaaagaccccaacgagaagcgcgatcacatggtcctgctggagttcgtgaccgccgccgggatcactctcggcatggacgagctgtacaagtaagtcgacaccaagcttccacagcaagcgaaccggaattgccagctggggcgccctctggtaaggttgggaagccctgcaaagtaaactggatggctttcttgccgccaaggatctgatggcgcaggggatcaagatctgatcaagagacaggatgaggatcgtttcgcatgattgaacaagatggattgcacgcaggttctccggccgcttgggtggagaggctattcggctatgactgggcacaacagacaatcggctgctctgatgccgccgtgttccggctgtcagcgcaggggcgcccggttctttttgtcaagaccgacctgtccggtgccctgaatgaactgcaggacgaggcagcgcggctatcgtggctggccacgacgggcgttccttgcgcagctgtgctcgacgttgtcactgaagcgggaagggactggctgctattgggcgaagtgccggggcaggatctcctgtcatctcaccttgctcctgccgagaaagtatccatcatggctgatgcaatgcggcggctgcatacgcttgatccggctacctgcccattcgaccaccaagcgaaacatcgcatcgagcgagcacgtactcggatggaagccggtcttgtcgatcaggatgatctggacgaagagcatcaggggctcgcgccagccgaactgttcgccaggctcaaggcgcgcatgcccgacggcgaggatctcgtcgtgacccatggcgatgcctgcttgccgaatatcatggtggaaaatggccgcttttctggattcatcgactgtggccggctgggtgtggcggaccgctatcaggacatagcgttggctacccgtgatattgctgaagagcttggcggcgaatgggctgaccgcttcctcgtgctttacggtatcgccgctcccgattcgcagcgcatcgccttctatcgccttcttgacgagttcttctgacccggtacctcagcgctagcggagtgtatactggcttactatgttggcactgatgagggtgtcagtgaagtgcttcatgtggcaggagaaaaaaggctgcaccggtgcgtcagcagaatatgtgatacaggatatattccgcttcctcgctcactgactcgctacgctcggtcgttcgactgcggcgagcggaaatggcttacgaacggggcggagatttcctggaagatgccaggaagatacttaacagggaagtgagagggccgcggcaaagccgtttttccataggctccgcccccctgacaagcatcacgaaatctgacgctcaaatcagtggtggcgaaacccgacaggactataaagataccaggcgtttccccctggcggctccctcgtgcgctctcctgttcctgcctttcggtttaccggtgtcattccgctgttatggccgcgtttgtctcattccacgcctgacactcagttccgggtaggcagttcgctccaagctggactgtatgcacgaaccccccgttcagtccgaccgctgcgccttatccggtaactatcgtcttgagtccaacccggaaagacatgcaaaagcaccactggcagcagccactggtaattgatttagaggagttagtcttgaagtcatgcgccggttaaggctaaactgaaaggacaagttttggtgactgcgctcctccaagccagttacctcggttcaaagagttggtagctcagagaaccttcgaaaaaccgccctgcaaggcggttttttcgttttcagagcaagagattacgcgcagaccaaaacgatctcaagaagatcatcttattaaggggtctgacgctcagtggaacgaaaactcacgttaagggattttggtcatgagattatcaaaaaggatcttcacctagatccttttaaattaaaaatgaagttttaaatcaatctaaagtatatatgagtaaacttggtctgacagttaccaatgcttaatcagactagagcttccatccgcttgccctcatctgttacgccggcggtagccggccagcctcgcagagcaggattcccgttgagcaccgccaggtgcgaataagggacagtgaagaaggaacacccgctcgcgggtgggcctacttcacctatcctgcccggctgacgccgttggatacaccaaggaaagtctacacgaaccctttggcaaaatcctgtatatcgtgcgaattgatccaccgtgcggctgcatgaaatcctggccggtttgtctgatgccaagctggcggcctggccggccagcttggccgctgaagaaaccgagcgccgccgtctaaaaaggtgatgtgtatttgagtaaaacagcttgcgtcatgcggtcgctgcgtatatgatgcgatgagtaaataaacaaatacgcaaggggaacgcatgaaggttatcgctgtacttaaccagaaaggcgggtcaggcaagacgaccatcgcaacccatctagcccgcgccctgcaactcgccggggccgatgttctgttagtcgattccgatccccagggcagtgcccgcgattgggcggccgtgcgggaagatcaaccgctaaccgttgtcggcatcgaccgcccgacgattgaccgcgacgtgaaggccatcggccggcgcgacttcgtagtgatcgacggagcgccccaggcggcggacttggctgtgtccgcgatcaaggcagccgacttcgtgctgattccggtgcagccaagcccttacgacatatgggccaccgccgacctggtggagctggttaagcagcgcattgaggtcacggatggaaggctacaagcggcctttgtcgtgtcgcgggcgatcaaaggcacgcgcatcggcggtgaggttgccgaggcgctggccgggtacgagctgcccattcttgagtcccgtatcacgcagcgcgtgagctacccaggcactgccgccgccggcacaaccgttcttgaatcagaacccgagggcgacgctgcccgcgaggtccaggcgctggccgctgaaattaaatcaaaactcatttgagttaatgaggtaaagagaaaatgagcaaaagcacaaacacgctaagtgccggccgtccgagcgcacgcagcagcaaggctgcaacgttggccagcctggcagacacgccagccatgaagcgggtcaactttcagttgccggcggaggatcacaccaagctgaagatgtacgcggtacgccaaggcaagaccattaccgagctgctatctgaatacatcgcgcagctaccagagtaaatgagcaaatgaataaatgagtagatgaattttagcggctaaaggaggcggcatggaaaatcaagaacaaccaggcaccgacgccgtggaatgccccatgtgtggaggaacgggcggttggccaggcgtaagcggctgggttgtctgccggccctgcaatggcactggaacccccaagcccgaggaatcggcgtgacggtcgcaaaccatccggcccggtacaaatcggcgcggcgctgggtgatgacctggtggagaagttgaaggccgcgcaggccgcccagcggcaacgcatcgaggcagaagcacgccccggtgaatcgtggcaagcggccgctgatcgaatccgcaaagaatcccggcaaccgccggcagccggtgcgccgtcgattaggaagccgcccaagggcgacgagcaaccagattttttcgttccgatgctctatgacgtgggcacccgcgatagtcgcagcatcatggacgtggccgttttccgtctgtcgaagcgtgaccgacgagctggcgaggtgatccgctacgagcttccagacgggcacgtagaggtttccgcagggccggccggcatggccagtgtgtgggattacgacctggtactgatggcggtttcccatctaaccgaatccatgaaccgataccgggaagggaagggagacaagcccggccgcgtgttccgtccacacgttgcggacgtactcaagttctgccggcgagccgatggcggaaagcagaaagacgacctggtagaaacctgcattcggttaaacaccacgcacgttgccatgcagcgtacgaagaaggccaagaacggccgcctggtgacggtatccgagggtgaagccttgattagccgctacaagatcgtaaagagcgaaaccgggcggccggagtacatcgagatcgagctagctgattggatgtaccgcgagatcacagaaggcaagaacccggacgtgctgacggttcaccccgattactttttgatcgatcccggcatcggccgttttctctaccgcctggcacgccgcgccgcaggcaaggcagaagccagatggttgttcaagacgatctacgaacgcagtggcagcgccggagagttcaagaagttctgtttcaccgtgcgcaagctgatcgggtcaaatgacctgccggagtacgatttgaaggaggaggcggggcaggctggcccgatcctagtcatgcgctaccgcaacctgatcgagggcgaagcatccgccggttcctaatgtacggagcagatgctagggcaaattgccctagcaggggaaaaaggtcgaaaaggtctctttcctgtggatagcacgtacattgggaacccaaagccgtacattgggaaccggaacccgtacattgggaacccaaagccgtacattgggaaccggtcacacatgtaagtgactgatataaaagagaaaaaaggcgatttttccgcctaaaactctttaaaacttattaaaactcttaaaacccgcctggcctgtgcataactgtctggccagcgcacagccgaagagctgcaaaaagcgcctacccttcggtcgctgcgctccctacgccccgccgcttcgcgtcggcctatcgcggccgctggccgctcaaaaatggctggcctacggccaggcaatctaccagggcgcggacaagccgcgccgtcgccactcgaccgccggcgcccacatcaaggcaccctgcct

**Supplementary Table S1.** Plasmids used in this study.

| **Name** | **Brief description** | **Reference** |
| --- | --- | --- |
| pPG012 | Parent plasmid for making XRBS plasmids. Contains an MCS, p15A and pVS1 origins, *oriT,* and kanamycin resistance | This study |
| pJG1082 | Intermediate parent plasmid for making mScarlet-I-based XRBS plasmids | This study |
| pAB215 | Intermediate parent plasmid for making msfGFP-based XRBS plasmids | This study |
| pAB154 | mScarlet-I expression plasmid harboring FL_A XRBS | This study |
| pAB155 | mScarlet-I expression plasmid harboring T1_A XRBS | This study |
| pAB156 | mScarlet-I expression plasmid harboring T2_A XRBS | This study |
| pAB157 | mScarlet-I expression plasmid harboring T3_A XRBS | This study |
| pAB161 | mScarlet-I expression plasmid harboring FL_G XRBS | This study |
| pAB162 | mScarlet-I expression plasmid harboring T1_G XRBS | This study |
| pAB163 | mScarlet-I expression plasmid harboring T2_G XRBS | This study |
| pAB164 | mScarlet-I expression plasmid harboring T3_G XRBS | This study |
| pAB172 | mScarlet-I expression plasmid harboring CON_G XRBS | This study |
| pAB173 | mScarlet-I expression plasmid harboring CON_A XRBS | This study |
| pAB174 | mScarlet-I expression plasmid harboring CON_GT1 XRBS | This study |
| pAB175 | mScarlet-I expression plasmid harboring CON_GT2 XRBS | This study |
| pAB176 | mScarlet-I expression plasmid harboring CON_GT3 XRBS | This study |
| pAB177 | mScarlet-I expression plasmid harboring CON_GT4 XRBS | This study |
| pAB187 | mScarlet-I expression plasmid harboring KpnI_Spc XRBS | This study |
| pAB188 | mScarlet-I expression plasmid harboring EcoRI_Spc XRBS | This study |
| pAB189 | mScarlet-I expression plasmid harboring BamHI_Spc XRBS | This study |
| pAB190 | mScarlet-I expression plasmid harboring SacI_Spc XRBS | This study |
| pAB191 | mScarlet-I expression plasmid harboring Spc_T1 XRBS | This study |
| pAB192 | mScarlet-I expression plasmid harboring Spc_T2 XRBS | This study |
| pAB193 | mScarlet-I expression plasmid harboring GGT XRBS | This study |
| pAB216 | msfGFP expression plasmid harboring CON_G XRBS | This study |
| pAB217 | msfGFP expression plasmid harboring CON_GT4 XRBS | This study |
| pAB218 | msfGFP expression plasmid harboring T3_G XRBS | This study |
| pAB219 | msfGFP expression plasmid harboring GGT XRBS | This study |
| pAB230 | msfGFP expression plasmid with C-terminal 6His tag harboring CON_G XRBS | This study |
| pAB231 | msfGFP expression plasmid with C-terminal 6His tag harboring CON_GT4 XRBS | This study |
| pAB232 | msfGFP expression plasmid with C-terminal 6His tag harboring T3_G XRBS | This study |
| pAB233 | msfGFP expression plasmid with C-terminal 6His tag harboring GGT XRBS | This study |

**Supplementary Table S2.** Primers used in this study.

| **Name** | **Sequence** | **Notes** |
| --- | --- | --- |
| 1879 | cgcGGATCCccggtgagatctcgatcc | FOR: PlacT5-XRBS-GFP from pED021. Cloned into pPG012 |
| 1880 | CGCgtcgacttaCTTGTACAGCTCGTCCATGC | REV: PlacT5-XRBS-GFP from pED021. Cloned into pPG012 |
| 1897 | ggcTCTAGAaataattttgtttaactttaagaaggagatatacatATGGTGAGCAAGGGCGAG | FOR: mScarlet-I with FL_A XRBS from AddGene clone 85044 |
| 1898 | gctgAAGCttaCTTGTACAGCTCGTCCATGC | REV: mScarlet-I FL_A and most XRBS variants |
| 1936 | cgcTCTAGAtttaactttaagaaggagatatac | FOR: T1_A XRBS derivative |
| 1952 | cgcTCTAGAtttaagaaggagatatacatATGG | FOR: T2_A XRBS derivative |
| 1937 | cgcTCTagaaggagatatacatATGGTG | FOR: T3_A XRBS derivative |
| oAB377 | gcgTCTAGAaataattttgtttaactttaagaaggagGtatacatATG | FOR: FL_G XRBS derivative |
| oAB378 | gcgTCTAGAtttaactttaagaaggagGtatacatATG | FOR: T1_G XRBS derivative |
| 1958 | CGCTCTAGAtttaagaaggagGtatacatatggtg | FOR: T2_G XRBS derivative |
| 1955 | CGCTCTagaaggagGtatacatatggtgag | FOR: T3_G XRBS derivative |
| oAB439 | GCGtctagaTTAACTTTaggagAtatacatATG | FOR: CON_A XRBS derivative |
| oAB416 | CGCtctagaTTAACTTTAGGAGGTatacatATG | FOR: CON_G XRBS derivative |
| oAB441 | CGCtctagaTAACTTTAGGAGGTatacatATG | FOR: CON_GT1 XRBS derivative. |
| oAB442 | CGCtctagaAACTTTAGGAGGTatacatATG | FOR: CON_GT2 XRBS derivative. |
| oAB443 | CGCtctagaACTTTAGGAGGTatacatATG | FOR: CON_GT3 XRBS derivative. |
| oAB444 | CGCtctagaCTTTAGGAGGTatacatATG | FOR: CON_GT4 XRBS derivative. |
| oAB447 | CGCtctagaCTTTAGGAGGTacccatATGGTGAGCAAGGGCGAG | FOR: KpnI_Spc XRBS derivative. |
| oAB448 | CGCtctagaCTTTAGGAGGTgaattcATGGTGAGCAAGGGCGAG | FOR: EcoRI_Spc XRBS derivative. |
| oAB449 | CGCtctagaCTTTAGGAGGTggatccATGGTGAGCAAGGGCGAG | FOR: BamHI_Spc XRBS derivative. |
| oAB450 | CGCtctagaCTTTAGGAGGTgagctcATGGTGAGCAAGGGCGAG | FOR: SacI_Spc XRBS derivative. |
| oAB451 | CGCtctagaCTTTAGGAGGTataatATGGTGAGCAAGGGCGAG | FOR: Spc_T1 XRBS derivative. |
| oAB452 | CGCtctagaCTTTAGGAGGTatatATGGTGAGCAAGGGCGAG | FOR: Spc_T2 XRBS derivative. |
| oAB453 | CGCtctagaGGTatacatATGGTGAGCAAGGGCGAG | FOR: GGT XRBS derivative. |
| oAB454 | gcgTCTAGAaataattttgtttaactttaagaaggagatataCATatgAGTAAAGGTGAAGAGCTGTTCACCGGGGTG | FOR: Altering first four codons of msfGFP |
| oAB455 | cgcGGATCCaaTTGCCAgctggagcgccctctggTAAGGTtgggaagTCTAGAttaactttaggagGtatacatATGAGTAAAGGTGAAG | FOR: CON_G XRBS upstream of msfGFP |
| oAB456 | cgcGGATCCaaTTGCCAgctggagcgccctctggTAAGGTtgggaagTCTAGActttaggaggtatacatATGAGTAAAGGTGAAG | FOR: CON_GT4 upstream of msfGFP |
| oAB457 | cgcGGATCCaaTTGCCAgctggagcgccctctggTAAGGTtgggaagTCTAGAaggagGtatacatATGAGTAAAGGTGAAG | FOR: T3_G upstream of msfGFP |
| oAB458 | cgcGGATCCaaTTGCCAgctggagcgccctctggTAAGGTtgggaagTCTAGAggtatacatATGAGTAAAGGTGAAG | FOR: GGT upstream of msfGFP |
| oAB459 | CTGTGGAAGCTTggtGTCgac | REV: msfGFP from pAB215. |
| oAB466 | cgcGTCGACTTAGTGGTGATGGTGATGGTGACCTCCACCCTTGTACAGCTCGTCCATGC | REV: Incorporates a gly-gly-gly-His_6_ tag at the CT end of msfGFP |
